# Late post-transplant recurrence of an anti-myeloperoxydase antibody-associated vasculitis in a former double-positive patient: a case report

**DOI:** 10.3389/fimmu.2026.1723903

**Published:** 2026-02-04

**Authors:** Nessma Chenaf-Benabdelmoumene, Melchior Chabannes, Didier Ducloux, Jamal Bamoulid, Thomas Crepin, Stéphane Lang

**Affiliations:** 1 Université Marie et Louis Pasteur, Centre Hospitalier Universitaire (CHU) de Besançon, Service de Néphrologie-Dialyse-Transplantation, Besançon, France; 2 Université Marie et Louis Pasteur, Etablissement Français du Sang (EFS), Institut National de la Santé et de la Recherche Médicale (INSERM), RIGHT (UMR 1098), Besançon, France

**Keywords:** case report, double positive patient, Goodpasture disease, kidney transplantation, post-transplant vasculitis recurrence, anti-myeloperoxydase-associated vasculitis

## Abstract

Post-transplant anti-neutrophil cytoplasmic antibody (ANCA)-associated vasculitis (AAV) recurrence on the allograft is rare and, to our knowledge, has never been described in the situation of a previous double-positive vasculitis with both ANCA and anti-glomerular basement membrane (GBM) antibodies. We report an unusual case of anti-myeloperoxydase (MPO) antibody-associated vasculitis recurrence occurring 14 years after kidney transplantation following a double-positive anti-GBM and anti-MPO glomerulonephritis. The transplant induction regimen consisted of anti-thymocyte globulin, and initial maintenance therapy associated tacrolimus, mycophenolate mofetil, and corticosteroids. Mycophenolate mofetil was discontinued 4 months after transplantation due to persistent leukopenia, and tacrolimus was maintained along with corticosteroids. At 14 years post-transplantation, the patient presented with diffuse alveolar hemorrhage, acute kidney injury stage 1 of Kidney Disease Improving Global Outcomes, proteinuria, microhematuria, serous otitis media and anti-MPO antibodies resurgence, without detectable anti-GBM antibodies. A kidney allograft biopsy was performed and showed rare active crescents with severe chronic injuries. This pulmonary and renal involvement was attributed to an anti-MPO antibody-associated vasculitis recurrence. The induction treatment of the relapse consisted of methylprednisolone at 5 mg/kg and rituximab at 375 mg/m^2^ per week for 4 weeks. Renal function remained stable, urinary protein to creatinine ratio decreased from 0.9 g/g to 0.3 and 0.2 g/g at 6 and 12 months, respectively. Microhematuria resolved at 6 months and remained absent subsequently. Maintenance treatment was continued with rituximab every 6 months. According to the literature, post-transplant isolated AAV recurrence on the allograft remains exceptional, ranging between 0.003 and 0.076 per patient per year. To our knowledge, there are no reported cases of post-transplant anti-MPO-associated vasculitis recurrence in a patient with former anti-MPO and anti-GBM antibody-associated vasculitis. This case underlines the fact that AAV can recur late after transplantation in previously double-positive vasculitis patients. Thus, close monitoring of clinical and biological signs of recurrence is necessary in these patients. Because the pathophysiology of this atypical entity remains unclear, further trials are still necessary to highlight the underlying mechanisms of this particular auto-immune association and, more specifically, of isolated post-transplant AAV recurrence in double-positive patients to improve prevention and to elaborate more efficient immunosuppressive strategies.

## 1 Introduction

Double-positive patients (DPP) are characterized by a clinical presentation compatible with vasculitis associated with the finding of both circulating anti-glomerular basement membrane (GBM) and anti-neutrophil cytoplasmic antibodies (ANCA) (1, 2). Anti-GBM antibodies target the alpha 3 chain of collagen IV on the renal and pulmonary basement membranes, clinically leading to a pulmonary–renal syndrome known as Goodpasture syndrome, which is defined by a combination of alveolar hemorrhage and glomerulonephritis (3). ANCA, specifically anti-proteinase 3 (PR3) and anti-myeloperoxydase (MPO) antibodies, target lysosomal enzymes within leukocytes, resulting in neutrophil activation and necrotizing injuries of small vessels. Both anti-GBM antibody-mediated vasculitis and ANCA-associated vasculitis (AAV) with extracapillary glomerulonephritis can progress to end-stage renal disease (ESRD), ultimately requiring kidney transplantation.

Double seropositivity is uncommon, with an estimated incidence of 0.6 per million inhabitants (1, 2). The concurrence of both ANCA and anti-GBM antibodies in a Goodpasture disease has already been recognized as a specific entity with a first description in the 1980s (1, 4, 5). McAdoo et al. (1), in a retrospective multicentric analysis, underlined that up to 47% of anti-GBM-disease-diagnosed patients are double-seropositive. Another review found 33.7% of DPP among patients with anti-GBM vasculitides (2). Anti-MPO antibodies tend to be over-represented in DPP as opposed to anti-PR3 (1). Clinically, DPP share characteristics with both AAV (i.e., older age distribution and longer symptom duration before diagnosis) and anti-GBM antibody-associated vasculitis (i.e., severe renal involvement and a high frequency of diffuse alveolar hemorrhage at initial presentation) (1). Renal and pulmonary involvement are the two main manifestations, and pulmonary–renal syndrome is identified in 42.1% of DPP cases (2). Pathologically, most renal biopsies show a diffuse active crescentic glomerulonephritis, involving more than 50% to 85% of the glomeruli, with IgG glomerular basement membrane linear deposits, granular deposits, and/or pauci-immune aspects (2). Compared to patients with single-positive Goodpasture disease, DPP with both ANCA and anti-GBM antibodies tend to present with more fibrocellular or fibrous crescents and with more chronic injuries (sclerotic glomeruli, interstitial fibrosis, and tubular atrophy) (1). DPP can relapse over time. According to Kidney Disease Improving Global Outcomes (KDIGO) 2024 guidelines, relapse is defined as the occurrence of increased disease activity after a period of partial or complete remission. It can be divided into major or minor, with major relapses defined as life- or organ-threatening (6). In DPP, the relapse can be associated with an isolated elevation in the ANCA level or in both ANCA and anti-GBM antibody titers (2). A few symptomatic cases of DPP recurrences with single-positive anti-MPO antibodies or with double-positive anti-MPO and anti-GBM antibodies have been exceptionally described, but always occurring on native kidneys (1). To our knowledge, isolated AAV relapse on the allograft is already an unusual phenomenon (7), but symptomatic AAV relapse occurring on the allograft in a former DPP has never been described (1, 8).

We herein report the first case of a late post-transplant anti-MPO antibody-associated vasculitis recurrence in a former double-positive anti-MPO and anti-GBM patient with a Goodpasture disease, occurring 14 years after renal transplantation.

## 2 Case description

**Figure 1:**
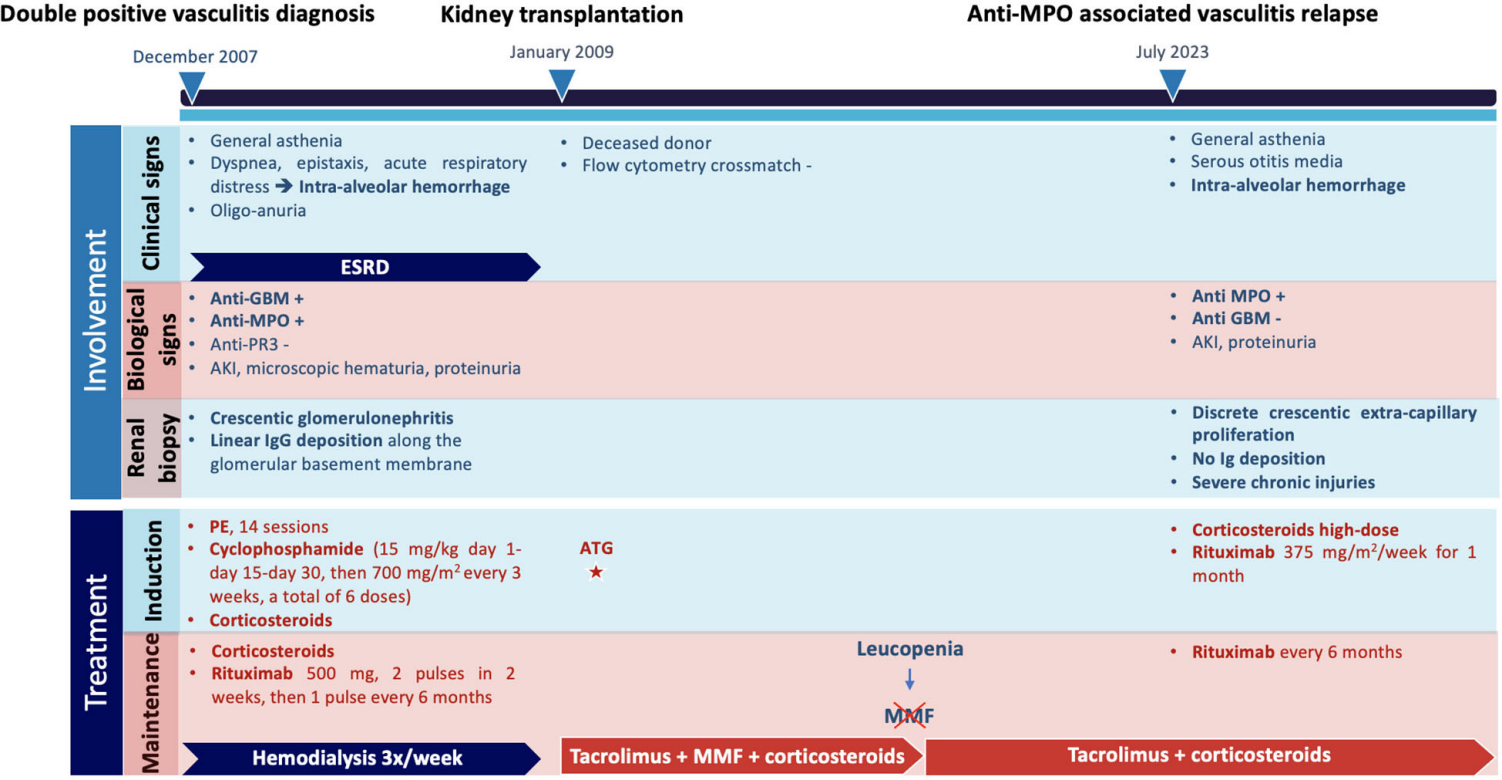
Timeline of the case report. ESRD, end-stage chronic renal disease; Anti-MPO, anti-myeloperoxydase antibody; Anti-GBM, anti-glomerular basement membrane antibody; Anti-PR3, anti-proteinase 3 antibody; AKI, acute kidney injury; IgG, immunoglobulin type G; PE, plasma exchange; ATG, anti-thymocyte globulin; MMF, mycophenolate mofetil.

The whole case report data are summarized in **Table 1** and in a synthetic timeline in **Figure 1**.

**Table 1:** Renal function and antibody titers’ evolution of the case report from December 2007 to July 2024.

Date	Anti-MPO antibody titer, IU/mL	Anti-GBM antibody titer, IU/mL	Creatininemia, µmol/L	eGFR, mL/min	uPCR, g/mmol	uRBCs/mm^3^
12/2007	**14.8** (*N* < 5)	**588** (*N* < 10)	926	–	–	–
2008	<5	<10	–	–	–	–
01/27/2009	<5	<0.8	–	–	0.09	424
04/2012	<5	<0.8	–	–	–	–
02/2013	<5	<0.8	–	–	–	–
02/2014	<5	<0.8	–	–	–	–
01/2015	<5	<0.8	–	–	–	–
01/2016	<5	<0.8	164	–	–	–
02/2017	<0.2	<0.8	150	27	0.02	<5
02/2018	–	<0.8	174	27	0.02	<5
04/2019	–	–	–	27	0.02	<5
01/2020	–	–	–	31	0.02	<5
01/2021	2.2	1.1	150	32	0.02	<10
01/2022	–	–	–	28	0.03	8
04/2022	–	–	181	25	0.02	4
07/2022	–	–	173	–	–	–
11/08/2022	**16** (*N* < 5)	<1.5 (*N* < 10)	211	20	0.06	69
11/28/2022	–	–	200	22	–	69
12/31/2022	**13** (*N* < 5)	–	189	23	0.03	39
02/2023	–	–	–	24	0.04	32
04/2023	–	–	175	25	0.06	8
07/09/2023	**277** (*N* < 20)	0.0 (*N* < 40)	219	20	0.09	424
07/21/2023	**355.8** (*N* < 20)	–	224	19	0.08	26
08/2023	**148.5**	–	191	23	0.07	–
09/2023	**45.8**	–	198	22	0.04	19
11/2023	–	–	241	12	0.02	<10
01/09/2024	**21.8**	–	218	–	0.04	2
01/16/2024	**25.0**	–	–	–	0.03	2
03/2024	14.9	–	201	22	0.03	–

December 2007: Goodpasture syndrome diagnosis with severe AKI, diffuse alveolar hemorrhage, significantly elevated anti-GBM antibodies, extra-capillary glomerulonephritis, and IgG deposition along the glomerular basement membrane on the renal allograft biopsy, associated with a positive anti-MPO antibody titer but without any active histological signs of AAV. November 2022: Community-acquired pneumonia episode, complicated with AKI, microhematuria, and proteinuria. Positive anti-MPO antibody titer (with a new threshold set by the hospital laboratory at *N* < 20) but without any other clinical signs of active AAV. July 2023: Late post-transplant anti-MPO-associated vasculitis relapse with AKI, microhematuria, proteinuria, diffuse alveolar hemorrhage, and a pauci-immune crescentic glomerulonephritis on the allograft biopsy, without any anti-GBM antibody titer increase.

Anti-MPO, anti-myeloperoxydase antibody; Anti-GBM, anti-glomerular basement membrane antibody; eGFR, estimated glomerular filtration rate; uPCR, urinary protein to creatinine ratio; uRBCs, urinary red blood cells; AAV, anti-neutrophil cytoplasmic antibodies-associated vasculitis; AKI, acute kidney injury. Bold values correspond to each value significantly superior to the threshold.

A 67-year-old woman with a prior medical history of arterial hypertension, active smoking, and no known history of connective tissue nor autoimmune disease was admitted to our department, in December 2007, for severe acute kidney injury (AKI) and diffuse alveolar hemorrhage. She had both highly elevated anti-GBM antibodies (588 IU/mL, *N* < 10) and a lower but significant anti-MPO antibody titer (14.8 IU/mL, *N* < 5). A native kidney biopsy showed a diffuse crescentic glomerulonephritis with linear IgG deposition along the glomerular basement membrane, with no chronic injuries. Given the low anti-MPO antibody titer with a typical histological pattern of anti-GBM glomerulonephritis, the diagnosis of Goodpasture syndrome was made. The patient was treated with plasma exchange, intravenous cyclophosphamide, and corticosteroids. Unfortunately, the kidney function rapidly deteriorated despite therapy, without subsequent recovery, requiring the initiation of hemodialysis a few days after the diagnosis. At 2 years later, in 2009, she underwent deceased-donor kidney transplantation. The immunosuppressive regimen consisted of anti-thymocyte globulin and methylprednisolone, followed by a maintenance therapy based on tacrolimus, mycophenolate mofetil, and corticosteroids. The post-transplantation period was unremarkable for 13 years, except for mycophenolate mofetil discontinuation at 4 months after transplantation due to leukopenia and persistent diarrhea. Both anti-MPO and anti-GBM antibodies remained negative until 2022.

In November 2022, the patient was admitted due to community-acquired pneumonia, complicated with AKI, microhematuria, and a urinary protein to creatinine ratio (uPCR) of 0.06 g/mmol. The anti-MPO antibody titer was increased (16 IU/mL, *N* < 5), but the patient did not present clinical signs of active AAV at this time. The infectious episode as well as the renal function resolved with ceftriaxone and rovamycine. The anti-MPO titer remained moderately elevated at 1 month later (13 IU/mL) (**Table 1**). The tacrolimus, as well as corticosteroid, levels remained stable within the target at the time of infection. In January 2023, she underwent a chest computed tomography (CT) scan, which was consistent with a bronchopneumonia.

**Figure 2:**
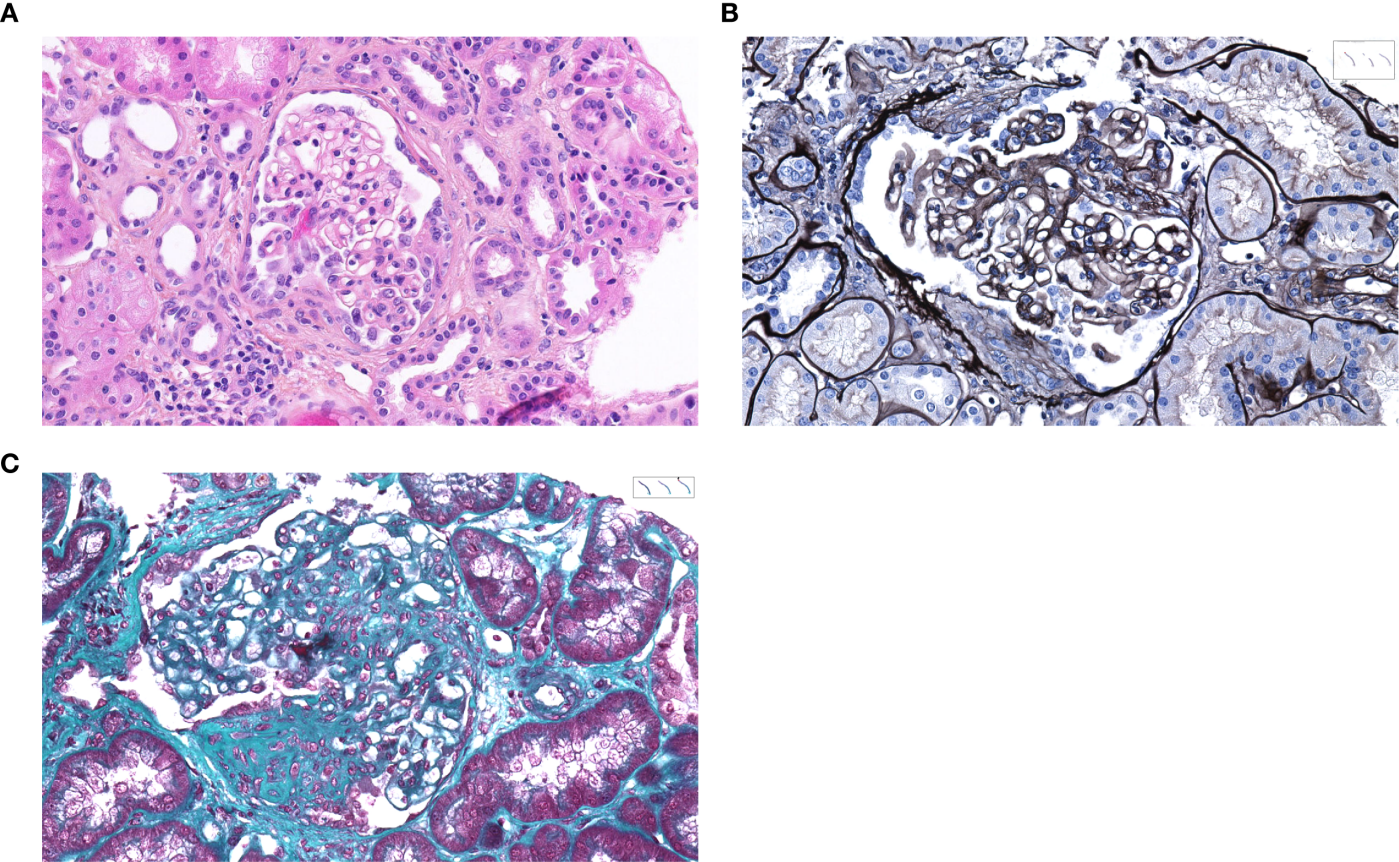
Kidney allograft biopsy snapshots at the time of post-transplant AAV relapse. **(A)** Hematoxylin and eosin stain. **(B)** Silver stain. **(C)** Masson’s trichrome stain. The kidney allograft biopsy showed one glomerulus with a segmental fibro-cellular crescent, associated with segmental mesangial fibrinoid necrosis.

In July 2023, she was admitted for the sudden onset of dry cough, asthenia, and chest and ear pain. The biological results showed AKI stage 1 with 224 µmol/L of creatinine (versus 175 µmol/L in April 2023), 0.09 g/mmol of uPCR, and microhematuria with 424/mm^3^ urinary red blood cells. There was an inflammatory syndrome with a C-reactive protein level of 354 mg/L and 9.97 G/L leukocytes. The patient remained afebrile. The serologic and molecular test results for aspergillosis, mucormycosis, legionellosis, pneumocystosis, *Mycoplasma*, cytomegalovirus, Epstein–Barr virus, and BK virus were negative. Multiplex viral PCR was likewise negative. Blood cultures remained sterile, and bacteriological as well as parasitological analyses of the bronchoalveolar lavage did not identify any pathogens. The hemoglobin level was normal at 13.8 g/dL. The chest CT scan was suggestive of diffuse alveolar hemorrhage, which was confirmed with the broncho-alveolar lavage (Golde score: 128). The anti-MPO antibody titer was significantly increased (355 IU/mL, with a new threshold set by the hospital laboratory at *N <*20), while the anti-GBM antibody titer remained negative. A kidney biopsy was performed on the allograft, and it showed limited crescentic glomerulonephritis with one cellular crescent in 17 glomeruli, without immune deposition on the immunofluorescence study (**Figure 2**). There were also severe chronic injuries with 50% of globally sclerotic glomeruli and 70% of interstitial fibrosis (**Figure 2**). According to the Berden classification (9), these findings were consistent with a sclerotic form of AAV glomerulonephritis. Thus, the diagnosis of post-transplant anti-MPO antibody-associated vasculitis relapse was retained. Consequently, a high-dose corticotherapy with methylprednisolone pulses was introduced, followed by oral prednisone and intravenous rituximab infusion.

All of the initial symptoms rapidly resolved after treatment initiation. During follow-up, the creatinine levels progressively returned to baseline with an estimated glomerular filtration rate (eGFR) of 23 mL/min/1.73 m^2^. At 6 months post-induction therapy, the uPCR levels decreased from 0.09 to 0.03 g/mmol, and microhematuria resolved (**Table 1**). The anti-MPO antibody titer significantly decreased (148 and 21 IU/mL at 1 and 6 months, respectively; *N* < 20) (**Table 1**). A chest CT scan conducted in January 2024 pointed out a decrease in the extent of ground-glass opacities, which are characteristic after-effects of alveolar hemorrhage. Following the intensification of the corticotherapy, the patient developed steroid-induced diabetes. She has also experienced several viral and bacterial infectious episodes, likely related to her immunosuppressive regimen. Notably, in March 2025, she was admitted to the intensive care unit for a septic shock due to a documented pulmonary and systemic adenovirus infection. More recently, in September 2025, she developed a *Campylobacter jejuni* infection, which resolved with a symptomatic treatment.

## 3 Discussion and conclusion

Although it is nowadays well established that ANCA and anti-GBM antibodies are antigenically distinct and that their co-existence in the same individual is not due to cross-reactivity, the underlying mechanism of their association is not fully understood (1). In our opinion, this case highlights several key points for those very unique patients.

Firstly, classifying accurately a double-positive vasculitis on a native kidney at presentation is not always an easy task—for example, our collective decision, made in 2007, to diagnose the case as a Goodpasture disease can be discussed. At that time, the specific entity of DPP had not yet been well described. Moreover, the absence of chronic injuries in the renal biopsy and the obvious histological pattern of anti-GBM-associated vasculitis, combined with the low anti-MPO antibody titer, can explain why it was identified as a typical Goodpasture syndrome. However, we can now suggest that a diagnosis of double-positive anti-MPO- and anti-GBM-associated vasculitis would have been more accurate. Indeed the native kidney biopsy showed granulomatous histiocytic inflammatory changes around ruptured glomeruli, which may indicate an inflammatory process mediated by a double-positive vasculitis, as well as numerous necrotic foci within the glomeruli, which were difficult to assess because they were retracted due to extracapillary proliferation. On the other hand, beyond the classification *per se*, the clinical disease severity at presentation seems to be comparable between single-positive anti-GBM cases and DPP, with 60% of patients requiring dialysis at presentation, and one-third involved by lung hemorrhage (1). Regarding renal and patient prognosis, death and progression to ESRD are similar between DPP and single-positive anti-GBM disease (1). As a consequence, rather than semantics *per se*, this suggests that anti-GBM disease is the predominant early disease phenotype in DPP (1). Nevertheless, in the case of DPP, the possibility of failing to identify ANCA positivity early represents a potential limitation, as it may influence the therapeutic response and the risk of relapse. Indeed, in terms of recurrence, single-positive anti-GBM disease relapse on native kidney is more frequent in conjunction with ANCA (1).

Secondly, in kidney transplants, AAV recurrence on kidney allografts should always be evocated, even if the presentation is not classical. Isolated AAV relapse in kidney transplant recipients is a rare phenomenon, occurring at about 0.02–0.03 per patient-year (6), and the average time from transplant to recurrence is 30.9 months, with a substantial variability from 15 days to 16 years (7). Because of the lack of information about post-transplant vasculitides recurrence in DPP, we can only highlight that DPP recurrence on native kidneys tends to occur late with an average time to first relapse at 4.4 years, with a substantial variability (range: 1.1–7.9 years), and predominantly involves anti-PR3 antibodies (1, 7). We herein report an isolated AAV relapse not only in a DPP but also on an allograft at 14 years after renal transplantation. Previous studies did not observe post-transplantation vasculitides recurrence in DPP in the short term (1), even less so in a single-positive presentation. Moreover, although single AAV recurrence rates are described as higher in patients with positive ANCA at transplantation (7), our patient was transplanted with a negative titer of both ANCA and anti-GBM antibodies. Regarding the clinical features of single-positive post-transplant AAV recurrence, there is a renal involvement in 60% cases, isolated or associated with other organs, and typically characterized by AKI with microscopic hematuria and proteinuria (7). Furthermore, the clinical presentation of post-transplant AAV recurrence may differ depending on the timing of the relapse. Masset et al. suggested that early recurrences can be more severe than late recurrences, which can be therefore more difficult to diagnose (10). Nevertheless, the diagnosis of AAV relapse made on the kidney allograft biopsy 14 years post-transplant can also be discussed. It showed 70% interstitial fibrosis and 50% global sclerosis, but only one cellular crescent in 17 glomeruli. We can suggest that there was also a part of chronic allograft injury and that a “grumbling” renal involvement was probably already present at the time of anti-MPO antibody elevation in November 2022, contributing to the degree of fibrosis. As with subclinical rejection, kidney graft biopsy should be readily considered in this type of case in order not to miss a low-grade inflammatory process.

Third, we have to pay attention to red flags. In our case, there was a time gap between the first post-transplant isolated increase of anti-MPO antibody titer in December 2022, occurring concurrently with community-acquired pneumonia, and the symptomatic AAV relapse diagnosed in July 2023. This time gap seems to be consistent with the pathophysiology of ANCA-associated vasculitides which are usually triggered by a current infectious context (11). Microscopic hematuria and low-grade proteinuria should have raised suspicion, but these findings were partly obscured by transplant-related confounding factors and intercurrent infection. This case underlines the high importance to systematically perform, especially in DPP with anti-GBM disease as initial predominant disease phenotype (1), a meticulous physical examination to track any sign consistent with a *de novo* or recurrent autoimmune involvement, completed by a frequent ANCA and anti-GBM antibody titer monitoring and even by a kidney biopsy. According to the KDIGO guidelines, the persistence of ANCA positivity, an increase in ANCA levels or a change in ANCA from negative to positive, may be predictive of future disease relapse and should be considered when making treatment decisions (6).

Finally, the immunosuppression regimen choice may be subject to debate. Following the early discontinuation of mycophenolate mofetil, given the low immunological risk, we have maintained, by consensus, since 2009, an immunotherapy regimen with tacrolimus and corticosteroids with a target trough level between 4 and 6 µg/L. To prevent the risk of AAV relapse, maintenance therapy with rituximab was initiated, although mycophenolate mofetil, methotrexate, or azathioprine were other possible alternatives. However, in the MAINRITSAN study (12), conducted in patients newly diagnosed with or relapsing AAV after induction with cyclophosphamide, rituximab was shown to provide better prevention of relapse than azathioprine at 5 years, whereas in the IMPROVE study (13), the relapse rates were higher with mycophenolate mofetil than with azathioprine. It should be underlined that our collective decision was limited by the fact that all of these trials were conducted in patients with native kidneys and not in kidney transplant recipients. However, this consensual choice of rituximab as a maintenance therapy could be debated in an elderly immunodepressed kidney transplanted patient. In fact, there are currently no clinical trials evaluating the benefit of continuing immunosuppressive therapy in patients with AAV who are not only elderly but also experiencing disease relapse and have undergone kidney transplantation. It should also be noted that the patient underwent a severe infectious complication. The duration of maintenance therapy, which has been proven to improve clinical outcomes in patients with native AAV-related glomerulonephritis, should be approached with greater caution in allograft recipients.

In conclusion, this first description of anti-MPO antibody-mediated vasculitis recurrence in a previously double-positive patient, occurring 14 years post-transplantation, underlines not only the possibility of relapse but also that it can recur on the allograft, late after renal transplantation. These results indicate the need for long-term monitoring of both ANCA and anti-GBM antibodies in these patients especially and thorough clinical assessment to screen for signs of autoimmune disease. Further trials are still required to highlight the underlying mechanisms of this singular autoimmune association, to improve its definition, and to enhance the development of more efficient and consensual therapeutic and preventive strategies.

## 4 Patient’s perspectives

The patient is regularly monitored in our transplant center. She is currently still under tacrolimus and prednisone (5 mg/day) with a stable renal function characterized by an eGFR of 21 mL/min/1.73 m^2^. Rituximab has been continued as maintenance therapy every 6 months since its initiation. The anti-MPO antibody titer remains above the positivity threshold, without any clinical signs of AAV.

## Data Availability

The original contributions presented in the study are included in the article/Supplementary Material. Further inquiries can be directed to the corresponding author.
